# Current News

**Published:** 1890-09

**Authors:** 


### Current News.

As Dr. R. B. Adair said in the Georgia State Dental Society, we cannot all be microscopists; we have to accept the evidences placed before us by conscientious scientific men, who, having demonstrated the facts to themselves, give us their positive testimony, throwing in such a flood of light that what was the most obscure subject we had to deal with now becomes the most clear and comprehensible.

---

Dr. B. H. Catching thinks no "dental exhibit" should be considered complete which does not include samples of the dental journals and copies of standard and recent publications, the literature of his profession being as essential to the true dentist as the instruments and appliances constituting the usual exhibit of dental associations.

---

Dr. Wardlaw says that his attention was first seriously drawn to the "germ theory" by the remarks of Dr. Sudduth after the display of photo-micrographs at the Louisville meeting.

---

A dentist's life is better worth living after he has learned to operate by looking in a mouth-mirror and given up bending over and under to see into the cavity. W. C. Browne.

---

Dr. B. H. Catching fills roots with oxide of zinc and boric acid, equal parts, mixed with chloride of zinc, a mixture which does not set rapidly, giving time for careful, deliberate work, and securing the lasting benefits of the acid.

---

Dr. W. C. Browne thinks that students who have graduated and passed the State Examining Board should at once be made members of their State Society without payment of any initiation fee. If thus identified with it from the beginning, they will probably always remain members. Otherwise they may drift away and perhaps never be heard of again.

